# Increased Inflammatory Markers Detected in Nasal Lavage Correlate with Paranasal Sinus Abnormalities at MRI in Adolescent Patients with Cystic Fibrosis

**DOI:** 10.3390/antiox10091412

**Published:** 2021-09-03

**Authors:** Jaehi Chung, Felix Wünnemann, Johanna Salomon, Sébastien Boutin, Dario L. Frey, Tobias Albrecht, Cornelia Joachim, Monika Eichinger, Marcus A. Mall, Mark O. Wielpütz, Olaf Sommerburg

**Affiliations:** 1 Division of Pediatric Pulmonology & Allergy and Cystic Fibrosis Center, Department of Pediatrics III, University of Heidelberg, Im Neuenheimer Feld 430, 69120 Heidelberg, Germany; Jaehi.Chung@med.uni-heidelberg.de (J.C.); Cornelia.Joachim@med.uni-heidelberg.de (C.J.); 2 Translational Lung Research Center Heidelberg (TLRC), German Center for Lung Research (DZL), 69120 Heidelberg, Germany; Felix.Wuennemann@med.uni-heidelberg.de (F.W.); johanna.salomon@web.de (J.S.); Sebastien.Boutin@med.uni-heidelberg.de (S.B.); DarioLucas.Frey@med.uni-heidelberg.de (D.L.F.); Tobias.Albrecht@med.uni-heidelberg.de (T.A.); Monika.Eichinger@med.uni-heidelberg.de (M.E.); Mark.Wielpuetz@med.uni-heidelberg.de (M.O.W.); 3 Department of Diagnostic and Interventional Radiology, University Hospital of Heidelberg, 69120 Heidelberg, Germany; 4 Department of Diagnostic and Interventional Radiology with Nuclear Medicine, Thoraxklinik at University Hospital of Heidelberg, 69126 Heidelberg, Germany; 5 Department of Infectious Diseases, Medical Microbiology and Hygiene, University Hospital of Heidelberg, 69120 Heidelberg, Germany; 6 Department of Otorhinolaryngology, Head and Neck Surgery, University Hospital of Heidelberg, 69120 Heidelberg, Germany; 7 Department of Pediatric Pulmonology, Immunology and Critical Care Medicine and Cystic Fibrosis Center, Charite-Universitätsmedizin Berlin, 13353 Berlin, Germany; marcus.mall@charite.de; 8 Berlin Institute of Health (BIH), 10178 Berlin, Germany; 9 German Center for Lung Research (DZL), Associated Partner Site, 13353 Berlin, Germany

**Keywords:** cystic fibrosis, inflammation, *Staphylococcus aureus*, chronic rhinosinusitis, paranasal sinus, magnetic resonance imaging

## Abstract

Chronic rhinosinusitis (CRS) is a characteristic feature of cystic fibrosis (CF) multiorgan disease and develops early in the life of patients with CF. The study aimed to correlate the inflammatory markers and the presence of structural abnormalities detected by MRI in the paranasal sinuses of patients with CF. Methods: Nasal lavage and MRI of the paranasal sinuses was performed in a cohort of 30 CF patients (median age 14 y; range 7–20 y). Morphological abnormalities characteristic of CF were evaluated with a dedicated CRS MRI scoring system and correlated with different inflammation parameters measured in nasal lavage. Inflammation of the paranasal sinuses was positively associated with structural abnormalities in MRI. The concentration of the pro-inflammatory markers neutrophil elastase (NE) and the neutrophil elastase/alpha1-antitrypsin (NE/A1AT) complex correlated significantly with CRS-MRI sum score (*p* < 0.05, *r* = 0.416 and *p* < 0.05, *r* = 0.366, respectively). *S. aureus* infection was associated with the increased pro-inflammatory cytokine activity of IL-6 and IL-8, and increased levels of NE/A1AT complex in our patients (*p* < 0.05, respectively). CRS-MRI sum score and individual sinus MRI scores were positively associated with inflammatory activity as a sign of CRS pathology present in CF.

## 1. Introduction

Cystic fibrosis (CF) is one of the most common hereditary diseases in the Caucasian population. Although it is a multiorgan disorder causing symptoms such as pancreatic insufficiency, intestinal malabsorption, infertility and abnormal sweat [1], the leading cause of morbidity and mortality is progressive lung destruction, characterized by mucus obstruction of the small airways, bronchiectasis, neutrophil-dominated inflammation and chronic bacterial infection [2]. The cystic fibrosis transmembrane conductance regulator (*CFTR*) gene encodes for a cAMP-regulated ion channel that is responsible for the adequate secretion of chloride and bicarbonate in epithelial cells and regulates the epithelial Na+ channel (ENaC) [3]. Mutations in the *CFTR* gene, resulting in decreased CFTR activity, cause impairments in the hydration of the airway’s epithelial surface with highly viscous mucus, which limits mucociliary clearance in the airways, leading to the infection and inflammation of airway tissues [4,5]. 

Besides chronic lung disease, CF is also characterized by upper airway affection, such as chronic rhinosinusitis (CRS), beginning in the first years of life [4,6]. It is believed that the highly viscous mucus in CF obstructs the ostia of the sinuses, leading to chronic bacterial infection and inflammation [7]. Chronic inflammation in the sinuses is believed to promote goblet cell hyperplasia and squamous cell metaplasia, causing remodeling processes [8,9]. The clinical symptoms of CRS in CF include nasal congestion, rhinorrhea, nasal polyps, anosmia and sleep disorder [10]. In patients with CF, paranasal sinuses can serve as niches for bacteria, such as *Staphylococcus aureus* (*S. aureus*), *Haemophilus influenzae* (*H. influenzae*) and *Pseudomonas aeruginosa* (*P. aeruginosa*), to inhabit the sinuses and evolve into highly pathogenic microorganisms [11,12,13,14,15], which, in unstable phases, may migrate and play a role in recurrent infections and the inflammatory processes of the lower airways [12,16]. 

However, the exact relationship of chronic inflammation in the upper airways with the development of structural abnormalities of the paranasal sinuses is not well understood. After bacterial colonization, IL-8 and TNF-α as chemoattractants induce the migration of neutrophils into the airways [17]. When stimulated, neutrophils release free radicals produced via NADPH or myeloperoxidase as a mechanism for killing pathogens [18,19]. In CF, neutrophil-dominated inflammation results in the overproduction of reactive oxygen species (ROS), leading to oxidative stress with increased lipid and protein peroxidation [20,21]. Additionally, large amounts of neutrophil elastase (NE) are released from azurophilic granules of the neutrophils [22]. NE has important protective functions, such as remodeling the extracellular matrix and cleaving pathogens [23]. Antiproteases, such as alpha1-antitrypsin (A1AT) and secretory leukocyte protease inhibitor (SLPI), are the physiological antagonists of NE and protect the airway tissues [23,24,25]. However, the permanent imbalance between oxidants and antioxidants in the inflammatory process in the paranasal sinuses of patients with CF leads to a situation in which irreversible structural damage to the surrounding tissues of the upper airways occurs. For the estimation of the inflammatory status of the paranasal sinuses in patients with CF, nasal lavage has been shown to be a good noninvasive and safe method for obtaining sufficient sample material [26,27,28].

The onset of the development of structural abnormalities in the paranasal sinuses of patients with CF appears very early, as the increased opacity of the paranasal sinuses in those patients can be seen on radiographs as early as 8 months of age [29]. In recent years, however, it has been shown that magnetic resonance imaging (MRI) of the head, with its excellent soft tissue differentiation capability, can quickly and reliably identify characteristic abnormalities of CRS, such as mucosal swelling, mucopyoceles, and nasal polyps in the sinuses of patients with CF [6,30]. Therefore, such an examination should be performed in patients with CF at regular intervals for diagnosis and therapy management.

To the best of our knowledge, the inflammation of the upper airways has not been studied in association with morphological changes via MRI of the paranasal sinuses in patients with CF before. Therefore, this pilot study aimed to correlate the presence of structural abnormalities and the CRS-MRI score of the paranasal sinuses with the inflammatory status measured in the nasal lavage supernatant in patients with CF.

## 2. Materials and Methods

### 2.1. Study Population

This project was conducted as a cross-sectional sub-study within a prospective longitudinal observational study in patients with CF at the CF center in Heidelberg, Germany. Informed written consent was obtained from the patients or their parents or legal guardians. Both the sub-study reported in the manuscript and the above-mentioned overarching prospective longitudinal observational study were approved by the ethics committee of the Medical Faculty of the University of Heidelberg (S-175/2020 and S-370/2011, respectively). Between February 2019 and February 2020, all CF patients of the CF Center Heidelberg who had received their annual check-up with clinical assessment, lung function tests, throat and nasal swabs, and, when possible, lavages and MRIs of the paranasal sinuses were included in the study. Age over 7 years was considered to be necessary for performing the nasal lavage correctly, according to the protocol. All CF patients received therapy according to the current guidelines. The exclusion criteria for this study were acute exacerbation at the time of the study visit (yearly check-up and/or MRI) or surgery performed on the paranasal sinuses or the nose within 24 months before the study. The MRI was performed on all patients on the same day of the yearly check-up, or within an interval up to 4 months before or after sampling the upper airway with nasal lavage and nose swabs. 

### 2.2. Sample Collection and Measurement of Inflammatory Parameters

Nasal swabs were collected in patients with CF from the anterior nares first for microbiological culture, and these were processed by the local microbiology laboratory according to German quality assurance guidelines for CF microbiology [31]. Afterwards, nasal lavage was performed in these patients with clinically stable disease. The nasal lavage fluid was obtained by rinsing 5 to 10 mL isotonic saline (0.9% NaCl, Braun, Melsungen, Germany) into each nostril, according to Beiersdorf et al. (2013) [26]. The patients were asked to hold the fluid for a few seconds in a reclined head position, then lean forward and exhale it. The fluid was collected in a sterile beaker. Lavage samples were centrifuged (300 rpm, 10 min, 4 °C). The supernatant was aliquoted and stored at −80 °C. Viable cells were pelleted (300 rpm, 10 min, 4 °C), resuspended and counted with a hemocytometer. Total protein concentration was measured using 25 μL of supernatant (Pierce BCA Protein Assay Kit, Thermo Fisher Scientific, Rockford, IL, USA) at a 562 nm wavelength. The concentration of the inflammation markers IL-1β, IL-6, IL-8 and TNF-α in the NL supernatant was determined with a bead-based immunoassay using flow cytometry (Cytometric Bead Arrays, BD Biosciences, San Diego, CA, USA). The NE/A1AT complex, SLPI and TIMP-1 were measured in duplicates using ELISAs (Human PMN-Elastase ELISA Kit, Invitrogen, Quantikine ELISA Human SLPI Immunoassay R&D Systems, Inc., Minneapolis, MN, USA, Quantikine Elisa Human TIMP-1 Immunoassay, R&D Systems, Inc., Minneapolis, MN, USA). The activity of free soluble neutrophil elastase was detected from the cell-free supernatant of nasal lavage probes with the FRET-based reporter NEmo-1 (Sirius Fine Chemicals, Bremen, Germany) [32,33]. All inflammatory mediators were normalized to the total protein concentration. 

### 2.3. MRI and Image Assessment

MRI of the sinuses from CF patients was performed on a clinical 1.5-T scanner (Magnetom Avanto, Siemens Healthineers, Erlangen, Germany) as previously described [6]. Native T1- and T2-weighted images were obtained. After the application of intravenous gadolinium-based contrast, T1-weighted images were acquired (Dotarem, Guerbet AG, Sulzbach, Germany). MRI scans were assessed with a CRS MRI scoring system evaluating all paranasal sinuses regarding sinus dimensions, degree of opacification, and specific abnormalities such as mucosal swelling, mucopyoceles, polyps and effusion [6]. Further, the deformation of the semilunar hiatus in the maxillary sinus was graded. The maximal CRS-MRI score is 68 [6]. 

### 2.4. Lung Function Test

Lung function variables were used to describe the clinical conditions of the patients in our cohort. The Forced Expiratory Volume in one second as a percent of the predicted value (FEV1%pred.) was determined with spirometry, and calculated according to reference values from the European Respiratory Society [34]. In addition, the lung clearance index (LCI), as a parameter to measure ventilation inhomogeneities in the small airways, was determined additionally with a multiple breath washout (MBW) test with N_2_ as a tracer gas [35,36]. The normal values for the LCI 2.5% (hereafter referred to only as LCI) we used are reported to range from 6.16 to 7.91 for school-aged children 6 to 18 years of age [37]. The higher the LCI value, the worse the airway ventilation due to mucus-related obstruction. 

### 2.5. Data Analysis

As part of the evaluation, the inflammation parameters measured in the nasal lavage should be evaluated together with the results of the MRI of the paranasal sinuses. For this purpose, the presence of certain sinonasal pathologies as well as the CRS MRI score should be correlated to individual inflammation parameters. Furthermore, the microbiological colonization of the upper airways as well as the results of the two lung function tests were included in the evaluation. The data were analyzed using GraphPad Prism 6 (Graph Pad software Inc., San Diego, CA, USA). The results are presented as mean ± standard deviation when distributed normally. Non-normally distributed variables were presented as median values with minimum and maximum values. The influence of the bacterial colonization on the inflammatory response was measured with the Mann-Whitney-U-test. The Spearman correlation coefficient was calculated for the correlation of inflammatory parameters and CRS-MRI scores, as well as single pathologies. A *p*-value below 0.05 was considered statistically significant. 

## 3. Results

**Figure 1:**
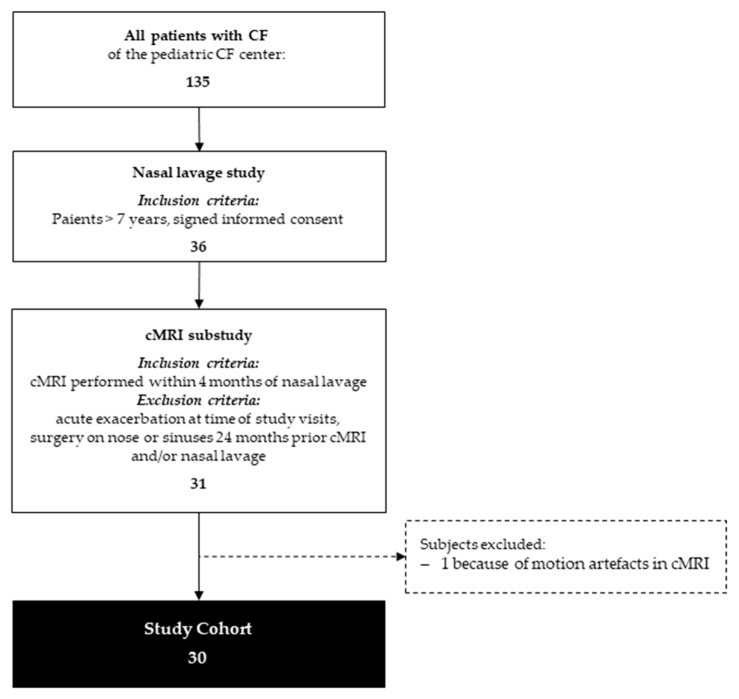
Patient recruitment.

This study was conducted as a sub-study of an ongoing longitudinal study, in which nasal lavage samples were taken to monitor inflammatory parameters. The recruitment of study participants from patients at our CF center is shown in Figure 1. At the time of the MRI sub-study, 36 of the 135 patients with CF from our CF center were participating in the longitudinal study performing nasal lavages. After reviewing the inclusion and exclusion criteria, 5 patients had to be excluded for the MRI sub-study, so 31 patients with cystic fibrosis (median age 14 years, range 7–20 years) in a stable clinical condition were included. Clinical symptoms of sinonasal disease were present in 17 of these 31. Of those 17 patients, 6 had undergone sinus surgery or resection of polyps, but this was more than 24 months before the start of the study. Due to motion artefacts, the MRI of one patient had to be excluded from the statistical analysis. Thus, in the end, 30 patients were included in this study. Although this is only a sub-cohort of all patients with CF, it mirrors a representative cross-section of patients from our CF center, with 12 patients homozygous for F508del, 14 patients heterozygous for F508del, and 4 patients with other *CFTR* mutations. For further information on clinical characteristics, refer to Table 1.

The upper airways of the CF patients in our cohort were vastly colonized with *Coagulase-negative Staphylococcus (CoNS)* (79.3%). In more than one third of the patients, the CF typical pathogen *S. aureus* (37.9%) was found in the upper airways. Only a few were colonized with *Corynebacterium species*, *Moraxella catarrhalis* or *Streptococcus species*. None of the patients with CF in our cohort were colonized with *P. aeruginosa* or *H. influenzae* in the nasal swabs. Further information on the bacterial colonization in our cohort can be found in the online supplement in Table S1. 

### 3.1. Inflammation Measured in Nasal Lavage 

We were able to obtain sufficient material in the nasal lavage samples from all patients in this study. The patients showed a varying concentration of vial cells, ranging from 1750 to 772,500 cells/mL. Accordingly, different amounts of total protein were shown, but the quantities were always sufficient to perform the determination of the inflammatory parameters. A complete overview of the results on the inflammatory parameters of the 30 patients with CF in the study is shown in Table 2. 

### 3.2. Influence of Microbiological Colonization in the Iinflammatory Response

**Figure 2:**
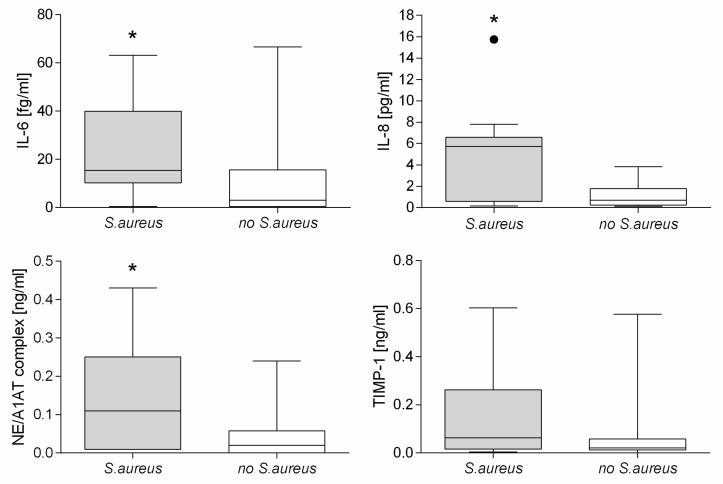
Comparison of the groups of patients with CF colonized with and without *S. aureus* for the inflammatory parameters IL-6, IL-8, the NE/A1AT complex and TIMP-1. Cytokine levels of IL-6 and IL-8, as well as of the NE/A1AT complex, were found to be significantly increased in patients with CF with colonization of *S. aureus* (* *p* < 0.05, ∙ outlier).

The results of nasal microbiological swabs corresponding to the time of nasal lavage were available for 29 of the 30 patients included in the study. Colonization with *S. aureus* was associated with higher inflammation. In patients with *S. aureus* infection of the upper airways, pro-inflammatory cytokines, such as IL-8 (*p* = 0.037) and IL-6 (*p* = 0.021), and also the NE/A1AT complex (*p* = 0.042), were significantly higher than in patients without. However, other proinflammatory cytokines and antiproteases, such as SLPI or TIMP, were also increased, but this difference was not statistically significant. The results for the cytokine levels of IL-6 and IL-8 as well as for the NE/A1AT complex and TIMP-1 are shown in Figure 2. 

For clarity, Table S1 in the online supplement lists the individual results of the 29 patients considered here for nasal bacterial colonization, CRS-MRI score, and inflammatory parameters.

### 3.3. Sinus Abnormalities Detected by MRI

**Figure 3:**
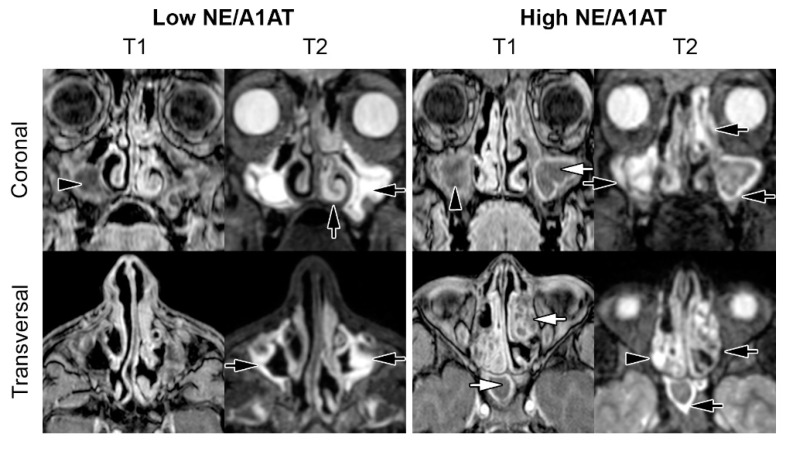
Representative MRI of the paranasal sinuses in patients with CF with low and high NE/A1AT ratios, respectively. A 16-year-old CF patient with a low NE/A1AT ratio demonstrated mucosal swelling (black arrows) of the maxillary sinus and left inferior concha, accompanied by polyps (black arrowheads) in the right maxillary sinus. The CRS-MRI score was 9. The 14-year-old CF patient with high NE/A1AT ratio also showed ubiquitous and more extensive mucosal swelling, polyps, as well as mucopyoceles (white arrows) of the maxillary, ethmoid and sphenoid sinus. The CRS-MRI score was 40. Abbreviations: NE = neutrophil elastase, A1AT = alpha1-antitrypsin.

Mucosal swelling was highly prevalent in the maxillary (95%), sphenoid (87%) and ethmoid sinuses (100%). Mucopyoceles were observed in the maxillary (82%), sphenoid (60%) and ethmoid sinuses (58%). Wall deformities of the maxillary sinus could be detected in 77% of patients in our cohort. In nine patients of our cohort, either one or both frontal sinuses were not developed. Figure 3 provides an overview of the typical pathologies found in the patients with CF in our study cohort. Table 3 provides an overview of the sinus pathologies seen via MRI in our study participants.

### 3.4. Inflammatory Parameters and MRI Scores 

**Figure 4:**
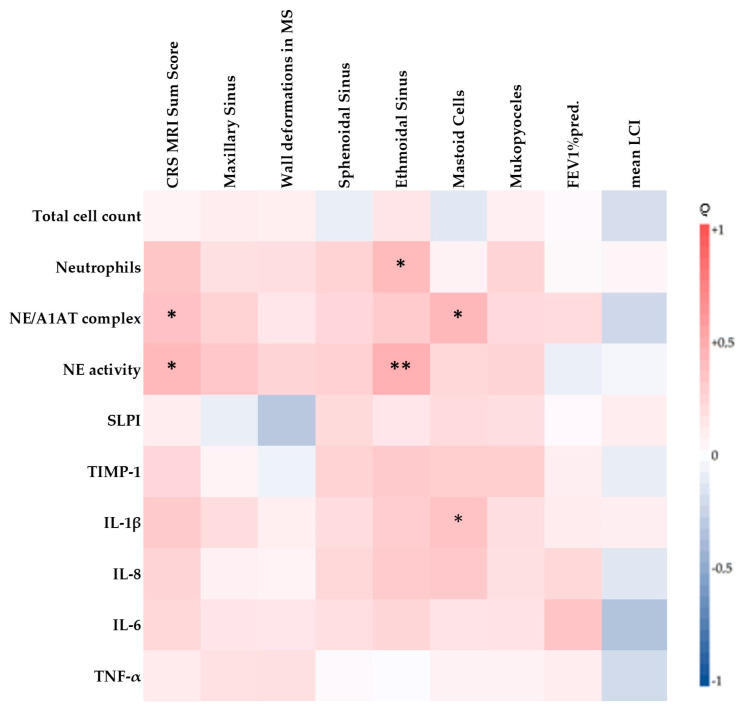
Spearman correlation of inflammatory parameters (NE/A1AT complex, SLPI, TIMP-1, IL1β, IL-6, IL-8, TNF-α) to MRI-scores of the sinuses and clinical parameters (pathogen colonization and lung function (FEV1%pred. and mean LCI)). * *p* < 0.05; ** *p* < 0.01. Abbreviations: MS = maxillary sinus.

The CRS MRI sum score correlated to all measured inflammatory parameters in the nasal lavage. Moreover, it correlated significantly to the NE/A1AT complex (*p* < 0.05, *r* = 0.366) and to the neutrophil elastase activity (*p* < 0.05, *r* = 0.416). Additionally, it could be seen that higher CRS-MRI subscores were positively associated with an increase in inflammatory markers. The score of the ethmoidal sinus correlated significantly to the number of neutrophils (*p* < 0.05, *r* = 0.411) and to the neutrophil elastase activity (*p* < 0.01, *r* = 0.468). Greater inflammation led to more radiographic changes associated with CRS being detected in the sinuses. SLPI, as a protective antiprotease, correlated inversely with the presence of wall deformations in the maxillary sinus (*r* = −0.313), but not significantly. A complete overview of the correlations between the measured inflammation parameters and the MRI scores is shown in Figure 4.

### 3.5. Influence of Sinonasal CF Disease on Lung Function

The values for the FEV1%pred. in our patient cohort ranged from 25 to 121%. The median was 84%, indicating that the majority of patients had normal FEV1 values. LCI values ranged from 5.53 to 18.04, with a median of 8.46, slightly above the cutoff level for this age group. No significant relation has been seen in our patient cohort between lung function estimated by FEV1%pred. and mean LCI and the inflammatory markers measured in nasal lavage (Figure 4), or morphological changes seen in MR imaging. 

## 4. Discussion

Sinus involvement is part of the multiorgan disease in patients with CF. Radiologic abnormalities associated with sinus disease are known to start early in the lives of CF patients. In our study, we demonstrated that increased inflammatory parameters in nasal lavage specimens from patients with CF correlate with morphologic changes and structural abnormalities in the paranasal sinuses seen on MRIs performed at that time.

### 4.1. Inflammation and Infection of the Upper Airways

Our results show a significant increase in the inflammatory activity in the upper airways of patients with CF. The measured levels for the individual inflammatory parameters are, in principle, consistent with the results of previous studies measuring inflammatory markers in nasal lavages [26,27,38,39,40]. Nevertheless, our values for cytokines seem to be somewhat lower compared to others. One reason for this might be the younger age in our cohort compared, e.g., to that published by Hentschel et al. [27]. However, our results reflecting cytokine activity still differ significantly from values measured for healthy subjects [27]. In this context, it is interesting to note that the concentration of antiproteases measured in our cohort was higher than that measured by other authors. This may confirm our assumption that the younger patients in our study cohort were clinically more stable than the older patients previously studied in other cohorts [26,27,39]. Another explanation for the lower inflammatory response could be that colonization with pathogenic bacteria was not yet as pronounced in the patients in our cohort. 

In our cohort, 11 patients (38%) were colonized with the typical CF pathogen *S. aureus*, which was associated with higher levels of inflammatory cytokines, neutrophil elastase and protective antiproteases. The neutrophil-dominated immune response, with the release of pro-inflammatory cytokines, usually leads to high oxidative stress in the airways. High concentrations of NE, released by neutrophils, overwhelm the antiprotease capacity and contribute to the destruction of the airway’s surface cells by cleaving antiproteases and also digesting the extracellular matrix [41,42]. Further, the ROS produced by leukocytes during phagocytosis can inactivate A1AT, and as a result interfere, in the protease/antiprotease ratio [43,44]. However, in patients in whom *Corynebacterium* sp. (*n* = 7, 24%) was detected in the upper respiratory tract, this colonization did not lead to such an inflammatory reaction as we had seen for *S. aureus*. Most of our patients (79%) were colonized with *CoNS*. Colonization with *CoNS* showed no significant influence on all of the inflammatory parameters, highlighting its role as a commensal bacterium, as also seen in other studies [45]. None of our patients were colonized with *P. aeruginosa* or *H. influenzae.* This also distinguishes our cohort from older patients with CF, in whom upper airway colonization was previously studied in relation to inflammatory activity [13,15]. 

In general, inflammatory responses in the upper airways, as seen in our data for *S. aureus*, can also lead to irreversible tissue damage, and reduced lung function in the lower airways if the airways are thought of as a single organ. However, in our cohort, no association was found between upper airway inflammation and lung function as a global parameter for the condition of a CF patient. This was true not only for FEV1%pred., but also for LCI, which reflects ventilatory inhomogeneities due to mucus in the airways. Future studies will show whether there really is no correlation, or whether it could not be demonstrated because of our study design or the relatively healthy cohort.

### 4.2. Inflammation and Structural Abnormalities in the MRI 

In a recent paper published by our group, it could be shown that the prevalence and severity of MRI abnormalities associated with CRS in CF increase with age. Accordingly, the CRS-MRI scores obtained in our present cohort were higher than in the cohort previously described in patients with CF ranging from 0 to 6 years of life [6]. Other previously published studies on patients with CF using CT reported an increased prevalence of aplasia, especially of the frontal sinuses, and hypoplasia of the maxillary and the sphenoid sinus in adolescent and adult patients with CF [46,47,48]. In the present study, we can confirm a higher rate of aplasia and hypoplasia of the frontal sinus in our patient cohort. However, the delays in sinus development in patients with CF remain poorly understood. According to our previously published data, mucosal swelling is the earliest sign of CF-related sinus disease, and is detectable as early as the first few months of life [6]. Mucosal swelling likely reflects inflammation, similarly to wall thickening of the lower airway, and may be reversible [49,50]. In addition, mucopyoceles seem to be a characteristic feature of CF and, when present, also dominate the morphologic changes in the paranasal sinuses. Our current results also demonstrate that inflammation of the paranasal sinuses may influence the structural changes characteristic of CRS, as the CRS-MRI subscores of all sinuses and the CRS-MRI sum score correlated positively with the measured inflammation parameters. This suggests that the higher the inflammation, the greater the morphologic changes in the paranasal sinuses. Previous studies found *P. aeruginosa* in the aspirates of mucopyoceles from the maxillary sinus [46,48]. Similar to lower respiratory tract mucus plugging, mucus retention in the paranasal sinuses may serve as a nidus for chronic infections and a reservoir for CF pathogens. In our present cohort of mostly school-aged CF patients, no nasal colonization with *P. aeruginosa* could be detected; however, those patients colonized with *S. aureus* showed significantly higher inflammatory parameters as well, and a trend, albeit not significant, toward an increased CRS-MRI sum score. The CRS-MRI sum score as well as the CRS-MRI subscore of the ethmoid sinus were positively associated with NE activity. This might indicate that the higher protease activity is related to destruction of the surrounding tissues of the corresponding paranasal sinuses. In this respect, it is likely that the colonization of the paranasal sinuses with typical CF pathogens triggers inflammation and oxidative stress, and that the structural abnormalities of the paranasal sinuses influence each other. Nasal polyps, also typical of cystic fibrosis, represent irreversible structural abnormalities caused by the benign hyperproliferation of chronically inflamed mucosal tissue, and may also be discussed in this context.

### 4.3. Limitations

Our study has several limitations. First, our cohort consists of patients with CF aged 7 to 20 years. With a median age of 14 years, it consists mainly of adolescents, and may relatively healthy compared with cohorts of previous studies. Second, because participation in the overarching longitudinal study performing nasal lavage was voluntary, not all patients with CF from our CF center who were eligible for this study participated. On the one hand, this could lead to a potential distortion of the study cohort, as it may not be as representative as originally thought. On the other hand, this may lead to a bias, as more therapy-adherent parents and patients may have consented to our sub-study, and thus influenced our cohort accordingly. Third, the cell and protein contents of our nasal lavage samples show significant interindividual differences. Although we can say, based on experience in determining inflammation parameters in our laboratory, that we remained above the minimum amount of material to be studied in each case, we obtained very low concentrations or activities in individual inflammatory parameters in some patients. Fourth, our study would have benefited if the subjective well-being of the participating patients had been additionally evaluated with a sinonasal outcome score (SNOT) [51]. However, to the best of our knowledge, the current most used SNOT-22 test [52] has not yet been validated for patients with CF. To assess treatment effects on CF-related CRS, especially of the new CFTR modulators, future studies evaluating an appropriate SNOT score are essential.

## 5. Conclusions

Inflammation of the paranasal sinuses is positively associated with structural abnormalities on MRI. The CRS-MRI sum score and the subscores for individual sinuses were positively associated with NE activity, suggesting that higher protease activity is related to CRS. We also found that upper respiratory tract infection with *S. aureus* was associated with a more pronounced inflammatory response. Therefore, our data may suggest that colonization with the CF-typical pathogen *S. aureus* upregulates inflammatory activity in the upper airways, thereby possibly influencing the morphological changes characteristic of CRS. Since this was a pilot study performed on a voluntary basis, further studies with a larger cohort and wider age range should be performed to evaluate the relation of infection, inflammation, and the pathogenesis of CRS in more detail. 

## Figures and Tables

**Table 1:** Clinical characteristics of the study population, *n* = 30.

Clinical Parameter	Median (Range)	Mean ± SD
Age (years)	14 (7–20)	13.7 ± 3.3
Sex, female, *n* (%)	10 (33.3%)	
BMI (kg/m^2^) (*n* = 29)	19.4 (14.6–24.1)	19.3 ± 3.0
*CFTR* genotype		
F508del/F508del, *n* (%)	12 (40.0%)	
F508del/other, *n* (%)	14 (46.7%)	
Other/other, *n* (%)	4 (13.3%)	
CFTR modulator therapy, *n* (%)	14 (46.7%)	
FEV1%pred. (*n* = 30)	83.7 (25.2–121.2)	84.5 ± 19.2
LCI (*n* = 28)	8.5 (5.5–18.0)	10.0 ± 3.4
Nasal swabs (*n* = 29)		
*Coagulase negative Staphylococcus*, *n* (%)	23 (79.3%)	
*Staphylococcus aureus*, *n* (%)	11 (37.9%)	
*Moraxella catarrhalis*, *n* (%)	2 (6.9%)	
*Corynebacterium* sp., *n* (%)	7 (24.1%)	
*Pseudomonas aeruginosa*, *n* (%)	0 (0%)	
*Haemophilus influenzae*, *n* (%)	0 (0%)	

Abbreviations: SD = standard deviation, BMI = body mass index, FEV1%pred. = forced expiratory volume in one second in percent predicted, LCI = lung clearance index, sp. = species. If a dataset was incomplete for a certain parameter, the number of patients for the respective parameter is given in parenthesis.

**Table 2:** Inflammatory parameters, *n* = 30.

Inflammatory Parameter	Median (Range)
Total Cells/mL	14,500 (1750–772,500)
Neutrophils (%)	82.61 (0–100)
Total protein level (µg/mL)	155.5 (11.76–415.11)
IL-1β (pg/mL)	1.78 (0.10–96.64)
IL-6 (fg/mL)	424.4 (0.0–68,392.53)
IL-8 (pg/mL)	91.04 (21.19–1270.54)
NE/A1AT complex (ng/mL)	2.89 (0–50.58)
NE activity (ng/mL)	0 (0–0.04)
SLPI (ng/mL)	192,337.48 (14.60–2,217,601.86)
TIMP-1 (ng/mL)	3.93 (0.02–51.35)

Abbreviations: IL = interleukin, TNF = tumor necrosis factor, NE = neutrophil elastase, A1AT = alpha-1-antitrypsin, SLPI = secretory leukocyte protease inhibitor, TIMP-1 = tissue inhibitor of metalloprotease-1.

**Table 3:** Sinus pathologies seen in MRI, *n* = 30.

	Patients with Sinonasal Pathologies, *n* (%)	CRS-MRI Score Median (Range)
**CRS-MRI sum score**		30.5 (9–44)
**Maxillary sinus**		13 (3–18)
Wall deformation	28 (93.3%)	2 (0–4)
Mucopyoceles	26 (86.7%)	1 (0–4)
Mucosal swelling	29 (96.7%)	3 (0–4)
Effusion	2 (6.7%)	0 (0–2)
Polyps	17 (56.7%)	1 (0–3)
**Sphenoidal sinus**		8.5 (0–12)
Mucopyoceles	21 (70%)	2 (0–4)
Mucosal swelling	30 (100%)	3 (0–3)
Effusion	0 (0%)	0 (0)
Polyps	0 (0%)	0 (0)
**Ethmoidal sinus**		8.5 (6–12)
Mucopyoceles	18 (60%)	2 (0–4)
Mucosal swelling	30 (100%)	4 (2–4)
Effusion	0 (0%)	0 (0)
Polyps	0 (0%)	0 (0)
**Mastoid cells**		0 (0–5)
Mucopyoceles	0 (0%)	0 (0)
Mucosal swelling	3 (10%)	0 (0–2)
Effusion	1 (3.3%)	0 (0–2)
Polyps	0 (0%)	0 (0)

## Data Availability

Data are contained within the article and Supplementary Materials.

## Supplementary Materials

The following is available at https://www.mdpi.com/article/10.3390/antiox10091412/s1, Table S1: An overview of the bacterial colonization of the study population with the corresponding CRS MRI sum score, *n* = 29.
