# Supplementary material for: Increased Inflammatory Markers Detected in Nasal Lavage Correlate with Paranasal Sinus Abnormalities at MRI in Adolescent Patients with Cystic Fibrosis
Source: Antioxidants (Basel). 2021 Sep 3;10(9):1412. doi: 10.3390/antiox10091412 (PMC8465538; doi:10.3390/antiox10091412)
Supplement: Supplementary file 1 [file antioxidants-10-01412-s001.zip › antioxidants-1330925-supplementary.pdf]

Online Supplement.

Table S1. An overview of the bacterial colonization of the study population with the corresponding CRS MRI sum score, *n* = 29.

	CRS MRI sum score	Total cellcount (cells/mL)	Total protein level (µg/ml)	NE/A1AT (ng/ml)	IL-8 (pg/ml)	IL-6 (fg/ml)	S. aureus	non pathogen (CoNS, Rothia, Corynebacterium, alpha-hemolytic streptococcus)	other/oppportunistic pathogen (C. freundii, Klebsiella, Moraxella catarrhalis)
Patient 1	9	11.750	246,36	0,94	66,14	49,35		x	
Patient 2	19	11.600	29,19	0,65	81,88	446,74		x	
Patient 3	20	11.000	224,49	5,48	156,65	282,61		x	
Patient 4	20	22.000	415,11	6,27	193,71	23,83		x	
Patient 5	20	8.250	221,54	1,87	119,51	1612,77		x	
Patient 6	20	18.000	17,61	2,15	115,81	611,16	x	x	
Patient 7	20	13.250	101,33	11,17	332,45	6754,27		x	
Patient 8	22	246.750	380,74	41,90	287,38	3909,81	x	x	
Patient 9	25	32.500	49,62	1,69	33,61	257,82			
Patient 10	25	5.750	255,74	5,03	49,05	350,48		x	x
Patient 11	26	3.250	214,07	2,61	45,72	3081,36	x	x	
Patient 12	28	19.000	346,36	0,33	49,9	0		x	
Patient 13	28	3.500	262,51	0,00	27,97	138,9		x	
Patient 14	30	41.500	226,57	1,18	132,92	378,05	x	x	
Patient 15	31	15.500	133,86	0,18	21,19	29,76		x	
Patient 16	31	6.000	28,43	0,80	37,03	215,05		x	
Patient 17	32	82.500	253,14	4,54	330,77	402,13		x	
Patient 18	32	29.750	77,02	5,91	294,74	1668,26		x	x
Patient 19	33	50.250	11,76	5,11	73,32	743,04	x	x	x
Patient 20	34	32.000	93,10	4,83	258,87	1.070,09	x	x	
Patient 21	35	22.250	110,21	20,53	630,52	2.635,41	x	x	
Patient 22	35	28.750	80,67	2,36	1.270,54	68392,53	x	x	
Patient 23	37	9.250	12,83	3,16	100,2	198,09	x		
Patient 24	38	3.000	177,09	0,36	44,51	144,89		x	
Patient 25	39	1.750	182,82	1,64	25,35	79,55	x		
Patient 26	40	13.500	36,77	8,77	64,73	609,73		x	
Patient 27	41	16.750	195,51	50,58	1.207,25	7.804,66	x		
Patient 28	42	772.500	93,95	12,80	173,98	2926,85		x	
Patient 29	44	7.500	43,43	2,17	69,61	197,56		x	x
